# Extracellular vesicle small RNA cargo discriminates non-cancer donors from pediatric B-lymphoblastic leukemia patients

**DOI:** 10.3389/fonc.2023.1272883

**Published:** 2023-11-13

**Authors:** Modeline N. Longjohn, Jo-Anna B. J. Hudson, Lourdes Peña-Castillo, Robert P. J. Cormier, Brandon Hannay, Simi Chacko, Stephen M. Lewis, Paul C. Moorehead, Sherri L. Christian

**Affiliations:** ^1^ Department of Biochemistry, Memorial University of Newfoundland, St. John’s, NL, Canada; ^2^ Beatrice Hunter Cancer Research Institute, Halifax, NS, Canada; ^3^ Discipline of Pediatrics, Memorial University of Newfoundland, St. John’s, NL, Canada; ^4^ Department of Biology, Memorial University of Newfoundland, St. John’s, NL, Canada; ^5^ Department of Computer Science, Memorial University of Newfoundland, St. John’s, NL, Canada; ^6^ Atlantic Cancer Research Institute, Moncton, NB, Canada; ^7^ Department of Chemistry & Biochemistry, Université de Moncton, Moncton, NB, Canada

**Keywords:** extracellular vesicles, leukemia, gene signature, small non-coding RNA, B-ALL

## Abstract

Pediatric B-acute lymphoblastic leukemia (B-ALL) is a disease of abnormally growing B lymphoblasts. Here we hypothesized that extracellular vesicles (EVs), which are nanosized particles released by all cells (including cancer cells), could be used to monitor B-ALL severity and progression by sampling plasma instead of bone marrow. EVs are especially attractive as they are present throughout the circulation regardless of the location of the originating cell. First, we used nanoparticle tracking analysis to compare EVs between non-cancer donor (NCD) and B-ALL blood plasma; we found that B-ALL plasma contains more EVs than NCD plasma. We then isolated EVs from NCD and pediatric B-ALL peripheral blood plasma using a synthetic peptide-based isolation technique (Vn96), which is clinically amenable and isolates a broad spectrum of EVs. RNA-seq analysis of small RNAs contained within the isolated EVs revealed a signature of differentially packaged and exclusively packaged RNAs that distinguish NCD from B-ALL. The plasma EVs contain a heterogenous mixture of miRNAs and fragments of long non-coding RNA (lncRNA) and messenger RNA (mRNA). Transcripts packaged in B-ALL EVs include those involved in negative cell cycle regulation, potentially suggesting that B-ALL cells may use EVs to discard gene sequences that control growth. In contrast, NCD EVs carry sequences representative of multiple organs, including brain, muscle, and epithelial cells. This signature could potentially be used to monitor B-ALL disease burden in pediatric B-ALL patients *via* blood draws instead of invasive bone marrow aspirates.

## 1 Introduction

B-lymphoblastic leukemia (B-ALL, formally known as B cell acute lymphoblastic leukemia) is the most prevalent pediatric malignancy in Canada and worldwide, which, together with T-cell acute lymphoblastic leukemia, make up 75 – 80% of all new pediatric cancers (1, 2). In B-ALL, developing B cells at different stages encounter developmental arrest, leading to abnormal growth and survival (3). Clonal proliferation of growth-arrested B cells, called lymphoblasts, in the bone marrow (BM) is soon followed by extramedullary migration and lymphoblasts circulating in the blood, which crowd out healthy cells (4, 5). Due to clonal expansion, the abundance of lymphoblasts compromises the lymphocyte population at immune sites, leading to impaired immune function.

In B-ALL, specific genetic aberrations at different stages of B cell development facilitate risk stratification and prognosis. There are two groups of common B-ALL-associated genetic abnormalities. The first is numerical abnormalities (hyperdiploidy and hypodiploidy) with loss or gain of chromosomes. In the second group, structural abnormalities (such as *E2A/TCF::PBX1*, myeloid/lymphoid or mixed-lineage leukemia [MLL] rearrangements, also known as KMT2A, *BCR::ABL1* [Ph-positive], *BCR::ABL1*-like and *ETV6::RUNX1*) contain translocations that encode chimeric proteins or move a gene close to a strong transcriptional promoter resulting in gene overexpression (6). Of these cytogenetic subtypes, the most favorable (low-risk) and most prevalent subtypes for pediatric ALL are *ETV6::RUNX1* and hyperdiploidy, while high-risk subtypes such as *BCR::ABL1*-like and *KMT2A*, which has low prevalence in pediatric patients > 1 year old, have the worst outcome (6). In addition to cytogenetic subtype-based risk stratification, other prognostic factors include patient age, central nervous system (CNS) involvement and response to induction/consolidation therapy (7).

The cure rates and survival outcomes, especially for pediatric B-ALL, have improved tremendously, largely due to molecular genetics, improved testing for measurable/minimal residual disease (MRD), risk-based treatment regimens, new targeted agents and allogeneic hematopoietic stem cell transplantation (HCT) (8). This has led to recent 5-year overall survival rates of up to 98% in standard risk patients and 75% to 92% 5-year event free survival in high risk patients (NCI high risk B-ALL (9), NCI standard risk B-ALL) (10). Despite the intense chemotherapy regimens, including CNS prophylaxis, approximately 20% of pediatric B-ALL patients suffer a relapse (11). Currently, the strongest independent prognostic factor of pediatric B-ALL relapse is MRD, which is low-level disease that is indicative of disease burden and is undetectable by conventional cytomorphology (12). MRD detection post-initial treatment correlates with poorer relapse-free survival (RFS) and overall surival (OS) (13). Specifically, MRD positivity during standard chemotherapy, especially after the end of consolidation, is associated with an increased risk of relapse; thus, early detection would enable salvage treatment to be initiated sooner, which could possibly improve treatment results (12, 14). Conventional methods for detecting MRD involve invasive bone marrow aspirates followed by polymerase chain reaction (PCR) and flow cytometry-based detection of abnormal lymphoblasts. However, less invasive methods to obtain samples to track therapy response and MRD could improve the frequency of monitoring of disease burden and enable real-time clinical decision-making.

Extracellular vesicles (EVs) are membrane-enclosed nanoparticles released by all living cells tested to date (15). Based on the size and mode of biogenesis, EVs are classified into three main groups, which significantly overlap in size and composition. Exosomes are 30-150 nm-sized EVs produced via the endocytic pathway (16). Microvesicles are 150-1000 nm-sized vesicles that are formed via the outward budding of the plasma membrane (17). Apoptotic bodies are 1000-5000 nm-sized particles produced during the final stages of apoptosis. During EV biogenesis, cell-derived biomolecules are shuttled into the EVs, resulting in bioactive cargo (proteins, lipids, metabolites and nucleic acids), with cargo content being representative of the originating cell (15). Thus, a unique and important characteristic of EVs is their cargo, which affects other aspects of EV biology, especially their function. For instance, EV surface protein cargo can act as ligands to interact with receptors on other cells and facilitate their uptake and downstream effects (15). In addition, EV miRNA content, especially when EVs are taken up by other cells, can stimulate other functions in the recipient (18). Hence, examining the cargo profile of EVs can be informative about the physiological or pathological state of the originating cell or the function of the EVs.

Nucleic acids that can be packaged into EVs include mRNA and microRNA (miRNA) (19, 20), long non-coding RNA (lncRNA) (21), as well as ribosomal RNA (rRNA) (19, 22). EVs have also been reported to carry genomic DNA (gDNA) and mitochondrial DNA (mtDNA); however, this has recently been challenged (23). The nucleic acid profile of cancer patient EVs has been used to identify signatures that distinguish them from non-cancer donors (NCD). Specifically, EV RNA signatures have been identified for cancers including, ovarian cancer (24), clear cell renal carcinoma (25) and breast cancer (26), acute myeloid leukemia (27), and chronic lymphocytic leukemia (28). To the best of our knowledge, the small RNA EV content of pediatric B-ALL has not been reported.

EVs hold the promise for filling the gaps in knowledge about pediatric B-ALL, such as how lymphoblasts prime environments such as the bone marrow to allow leukemogenesis and how disease progression can be monitored using less invasive techniques. Specifically, EV physical characteristics (size and concentration) and biological properties, especially RNA content, have shown promise as a biomarker and for understanding etiology in other diseases, including leukemias such as acute myeloid leukemia (AML) (29). In pediatric B-ALL patients, the most relevant biofluids that carry clinically actionable biomarkers are peripheral blood (PB) plasma, bone marrow aspirate and cerebrospinal fluid. In pediatric B-ALL PB plasma, the size and concentration of EVs have not previously been measured, especially in comparison to age-appropriate NCD. Furthermore, RNA transcript types such as miRNA and mRNAs, which have been previously linked to pediatric B-ALL, have not been clearly explored in pediatric B-ALL EVs. Therefore, we have explored pediatric B-ALL EVs from diagnostic samples, in comparison to NCD EVs, as a proof-of-principle study to determine if EVs could be a source for easily accessible biomarkers.

## 2 Materials and methods

### 2.1 Ethics statement

This study was approved by the Human Research Ethics Board of the Health Research Ethics Authority of Newfoundland and Labrador (protocol #2018.069). Samples obtained from the British Columbia Children’s Hospital biobank were collected under the protocol number # H13-03111, as approved by their local research ethics board. Written informed consent was obtained from all participants before enrollment. All methods were carried out according to the Canadian Research Tri-Council policy on ethical conduct for research involving humans (https://ethics.gc.ca/eng/policy-politique_tcps2-eptc2_2018.html).

### 2.2 Participants

**Figure 1:**
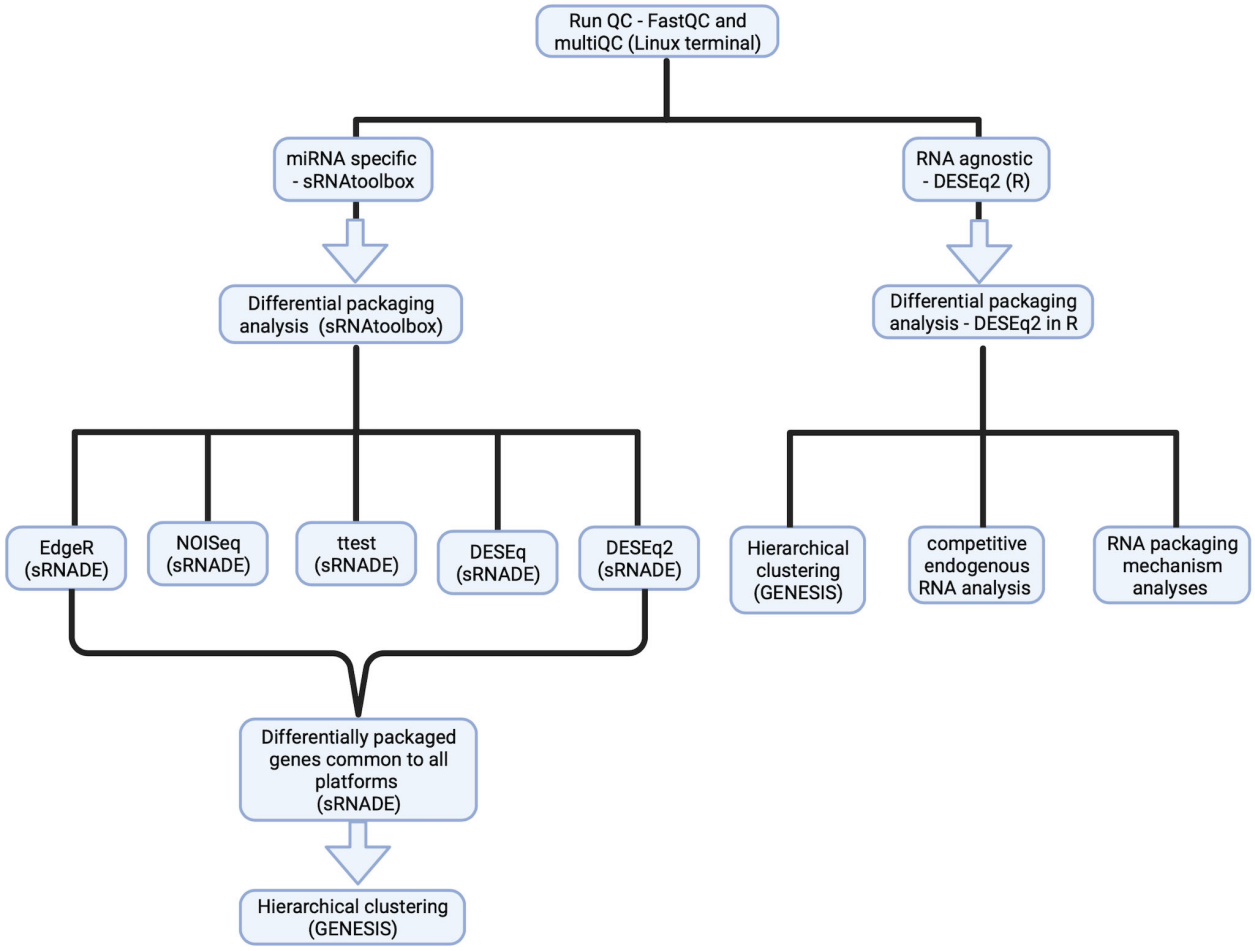
Experimental design for small RNASeq analysis to identify a B-ALL EV small RNA signature. Detailed steps for sample processing for RNASeq data analysis, using the miRNA-specific platform- SRNAtoolbox (left) for a miRNA-specific signature and the RNA agnostic platform – DESeq2 for an RNA agnostic signature (right).

PB plasma from NCD and pediatric B-ALL patients at diagnosis were obtained (**Table 1**) and used for experiments as outlined in **Figure 1**. Additionally, NCD PB plasma samples and conditioned media from pediatric B-ALL immortalized cell lines were used for optimizing EV isolation techniques.

**Table 1:** Characteristics of patient or non-cancer donor (NCD) blood plasma samples.

Sample ID	Sex	Age at collection (years)	Donor diagnosis	Source	PB WBC Count (x10^9^/L)	Plasma volume (µl)	EV small RNA (ng)	Read count
NCD1	Male	2	Spinal muscular atrophy	BCCH Biobank	NA^a^	1000	76.64	1.25E+07
NCD2	Male	9	Acute tonsilitis	BCCH Biobank	NA	1000	72.84	1.11E+07
NCD3	Male	3	Spinal muscular atrophy	BCCH Biobank	NA	1000	29.89	2.03E+07
NCD4	Male	17	Epilepsy	BCCH Biobank	NA	1000	18.89	2.35E+07
NCD5	Female	17	Epilepsy	BCCH Biobank	NA	1000	1.73	1.54E+07
NCD6	Female	12	Spinal muscular atrophy	BCCH Biobank	NA	1000	0.05	2.94E+07
2021PB07B_E	Male	11	*ETV6::RUNX1* pediatric B-ALL	Janeway	NA	1000	110.15	9.64E+06
2021PB08B_E	Male	9	*ETV6::RUNX1* pediatric B-ALL	BCCH Biobank	1.20	480	227.06	1.39E+07
2021PB09B_E	Male	3	*ETV6::RUNX1* pediatric B-ALL	BCCH Biobank	24.40	480	314.02	8.44E+06
2021PB10A_E	Female	3	*ETV6::RUNX1* pediatric B-ALL	BCCH Biobank	16.20	370	9.28	9.76E+06
2021PB12B_E	Male	2	*ETV6::RUNX1* pediatric B-ALL	BCCH Biobank	2.20	460	204.73	7.11E+06
2021PB13A_p	Female	12	*BCR::ABL*-like pediatric B-ALL	BCCH Biobank	507	290	457.22	1.93E+07
2021PB14B_p	Male	17	*BCR::ABL*-like pediatric B-ALL	BCCH Biobank	3.10	440	38.25	8.15E+06
2021PB15A_p	Female	17	*BCR::ABL*-like pediatric B-ALL	BCCH Biobank	49.90	320	1.56	1.16E+07

a NA, not available.

Human plasma from whole blood collected in EDTA tubes was obtained from the British Columbia Children’s Hospital biobank and the Janeway Children’s Hospital, Newfoundland and Labrador (non-biobanked samples). Whole blood was processed within 3 h of collection by centrifugation at 5000× g for 15 min at 4°C to separate the plasma fraction. The plasma fractions were stored at -80°C until use. Frozen plasma aliquots were slowly thawed on ice and treated for use as outlined in subsequent sections. Donors did not fast.

### 2.3 Cell culture

CCRF-SB (ATCC no.: CCL-120; Manassas, VA) was obtained from the American Type Culture Collection (ATCC). RCH-ACV (DSMZ no.: ACC 548, DSMZ, Germany) was obtained from the DSMZ, while UoC-B1 was a kind gift from the laboratory of Dr. Williams Evans from St. Jude’s Children’s Research Hospital, Memphis, Tennessee, USA. CCRF-SB, RCH-ACV and UoC-B1 were maintained in RPMI 1640 media (Gibco, Thermofisher, Cat. #: 11875093) supplemented with 10% heat-inactivated fetal bovine serum (FBS, Thermofisher, Cat. #: 16000044) and 1% penicillin and streptomycin (5,000 u/mL, Thermofisher Cat. #: 15070063) (RPMI complete media). Cell lines were cultured in their respective complete media at 37°C and 5% CO_2_. For EV isolation, cells grown to high density were washed with EV-depleted RPMI1640 and then cultured for 24h. EV-depleted media was generated as previously described (30). Briefly, 20% heat-inactivated FBS (RPMI-20%) was centrifuged at 100,000 × g for 18 h at 4°C in an SW-28 rotor (Beckman Coulter, Brea, CA) to deplete FBS derived vesicles, then filtered through a 0.22 µm filter and stored at 4 °C. FBS-free RPMI complete media was prepared using all ingredients except FBS. Vesicle-free media for culturing was prepared by mixing vesicle-free RPMI-20% and FBS-free RPMI-complete media in a 1:1 ratio. Cell-conditioned media (CCM) was then collected by centrifugation at 500 x g for 5 min followed by isolation as described below.

### 2.4 Peptide affinity-based isolation (Vn96) of EVs

EVs were isolated from CCM as previously optimized (31, 32) using Vn96 (Biosynth Ltd, UK). Vn96 is a 27-mer synthetic peptide that recognizes at least 5 unique heat shock proteins (HSPs) expressed on the surface of EVs (31, 33). For incubation, CCM from cell lines and lymphoblasts were centrifuged at 2,000 x g to pellet apoptotic vesicles and debris. CCM and plasma EVs were isolated using the Vn96 peptide ME kit for cell culture media and biofluids (Biosynth Ltd, UK) according to the manufacturer’s instructions. Briefly, CCM (at least 3.5 ml) was incubated with 60 µg (24 µl) at a concentration of 2.5 mg/ml and was incubated for 1 h at RT with 360° rotation. Plasma (**Table 1**) was diluted with 0.1 µm filtered sterile 1x phosphate-buffered saline (PBS) in a 1:1 ratio and incubated with 60 µg Vn96 (24 µl) at a concentration of 2.5 mg/ml for 1 h at room temperature with 360° rotation. Post incubation, CCM-Vn96 mix or diluted plasma-Vn96 mix was centrifuged at 17,000 g for 15 min at 4°C. The resulting translucent pellet was washed thrice in 0.1 μm filtered sterile 1x PBS supplemented with 1:1000 phenylmethylsulphonyl fluoride (PMSF) at 17,000 x g for 10 min at 4°C. The pellet was then resuspended in the appropriate buffer: 400 μl of mirVana lysis buffer for RNA isolation, 5X Laemmli sample buffer (Tris buffer pH 6.8, glycerol, β-mercaptoethanol, Bromophenol blue) for Western blot or 100 μl 1 mg/ml Proteinase K for Vn96-EV dissociation.

### 2.5 Nanoparticle tracking analysis of extracellular vesicles

NTA analysis was done as described (34). Briefly, NCD and B-ALL plasma were slowly thawed on ice and diluted at 1:1000 in 0.1-μm-filtered PBS. Diluted samples were immediately analyzed on a Nanosight NS300 with software version 3.4.003 (Malvern; UK). Five videos of 60 sec each were acquired using camera level 15 at a temperature of 25°C and syringe pump speed of 25 ml/s for all samples. Videos were analyzed using a detection threshold of 10 to obtain the mean diameter, mode diameter and mean number of particles/ml. The mean number of particles/ml was used to estimate the original concentration.

### 2.6 Transmission electron microscopy

For pediatric B-ALL cell lines, EV-Vn96 Pellets were resuspended in PBS and EVs dispersed from Vn96 peptide by digestion overnight with 25 µg proteinase K enzyme (Sigma-Aldrich) (31) at 37 °C. Post digestion, the Vn96-EV-proteinase-K mixture was centrifuged at 17,000 × g for 15 min to remove undigested material. Diluted dispersed EVs from cell lines and patient lymphoblast CCM were placed on formvar-carbon electron microscope grids (Electron Microscopy services, Hatfield, PA, USA) and dried for up to 60 mins. Grids were floated sample-side down in pyrogen-free water followed by fixation with 3.7% paraformaldehyde for 15 min and two washes with pyrogen-free water by slow dropwise application for 60 sec each time. Grids were then contrasted with 2% uranyl acetate (w/v) for 6 mins, followed by one additional water wash as above. All solutions were filtered using 0.1-µm syringe filters (4611; Pall Corp; Port Washington, NY). Dried grids were then viewed using a Tecnai Spirit transmission Electron Microscope (TEM) operating at 80KV (FEI; Hillsboro, OR).

### 2.7 Western blot

Cell pellets from cell lines were lysed in RIPA lysis buffer at a concentration of 1 x 10^6^ cells/100 µl in 1x RIPA lysis buffer supplemented with 2 μg/mL aprotinin (Sigma Aldrich, St. Louis, MO, USA), 1% phosphatase inhibitor cocktail (Sigma Aldrich, St. Louis, MO, USA), 1 mM sodium orthovanadate (New England Biolabs, Ipswich, MA, USA) and 1 mM PMSF (Sigma Aldrich, St. Louis, MO, USA). Blood plasma was lysed in the buffer as above, at a ratio of 1:1 plasma:2x RIPA lysis buffer (supplemented as above). Samples were then incubated on ice for 10 mins, followed by centrifugation at 17,000× *g* at 4°C for 10 min. The supernatant was stored at −80°C until further use. Vn96-EV pellets isolated as described above were resuspended in 0.1 µM filtered 1× PBS and mixed with 5X Laemmli sample buffer (300mM Tris buffer pH 6.8, 50% (v/v) glycerol, 25% β-mercaptoethanol, 0.05% (w/v) Bromophenol blue, 10% (w/v) sodium dodecyl sulphate) to a final concentration of 1x. Cell and EV lysates were separated under reducing conditions for all except CD63. For all markers except Calnexin and Albmin,1 ug for cell or plasma protein lysates were loaded, while 0.1 ug was loaded for Calnexin and Albumin. For EVs, proteins from 3 x 10^6^ cell derived EVs and 150 ul plasma were loaded. The proteins were separated using 10% and 12% SDS-PAGE as required. Proteins on gels were transferred onto nitrocellulose membranes, followed by blocking with 5% (w/v) skimmed milk in 0.1% TBST (NaCl, Tris base, 0.01% Tween-20). Antibodies were diluted as per the **Supplementary Methods**. Western chemiluminescent HRP substrate (Immobilon ECL Ultra Western HRP Substrate) was used for detection. Western blot image acquisition was done on a gel documentation system (Bio-Rad, Hercules, CA), followed by simple image manipulation involving only brightness and contrast adjustments of the entire image.

### 2.8 Total small RNA isolation

mirVana miRNA isolation kit (Ambion, Life Technologies, Carlsbad, CA, USA) was used for total small RNA extraction following the manufacturer’s instructions. The EV-Vn96 pellet was resuspended in lysis-binding buffer followed by vortexing. Next, miRNA homogenate additive was added, vortexed and incubated on ice. Post incubation, acid-phenol chloroform was added, followed by vortexing, and centrifugation for phasic separation. The top aqueous layer was then collected and incubated with 100% Ethanol, followed by deposition on a silica-based filter cartridge which was then centrifuged briefly. The total small RNA captured on the cartridge was washed thrice with proprietary washing buffer. Finally, the captured total small RNA was eluted in 100 µl RNAse-free water heated to 95°C and stored at - 80° for later use. Total small RNA in water was concentrated using the Savant speed vacuum centrifuge concentrator (Thermo Scientific, Asheville, NC) at 43°C for 2 rounds of 30 mins each. One µl RNA per well was then used for quantification using the Agilent bioanalyzer (Santa Clara, CA). Concentrated total small RNA samples were then sent to the Atlantic Cancer Research Institute (ACRI, Moncton, New Brunswick, Canada) for library preparation and sequencing.

### 2.9 RNA QC

The quality and quantity of the RNA were assessed using the Agilent small RNA kit (Agilent, 5067-1548) and the HS RNA assay on the fragment Analyzer (Agilent, 5067-5576) for integrity and size distribution respectively.

### 2.10 Library preparation

The library was prepared using 10ng RNA input or less with a library normalized for all samples and the NEXTFLEX Small RNA-Seq Kit v3 (Perkin Elmer) was used, following the manufacturer’s instructions. Reverse transcribed samples were subjected to a bead clean-up without any size selection. For the PCR enrichment, a different unique dual index (Perkin Elmer) was added to each sample, and 22 PCR cycles were used. Amplified libraries were purified without any size selection initially; however, due to high molecular weight templates, a final size selection was performed to obtain the final library. The quality of the library was assessed as follows; size distribution was determined with the D1000 assay on the Tapestation (Agilent). The KAPA Library Quantification Kit (Roche) was used to evaluate the concentration.

### 2.11 Small RNA sequencing

Equimolar amounts of libraries were first sequenced on the iSeq 100 instrument (Illumina) using single-end sequencing of 1X100 to assess both library and pooling qualities. Libraries’ inputs were rebalanced following Illumina’s recommendations to ensure an equal representation of each sample. Libraries were then sequenced using the Novaseq 6000 instrument (Illumina). Samples were loaded on an S1 flow cell, and a single-end sequencing of 1X101 was used.

### 2.12 Data processing

The data processing pipeline for this study is shown in **Figure 1**. Adaptor sequences were removed, and size filtered using Cutadapt (v4.0) (35), followed by QC using FastQC (v0.11.9) (36) and MultiQC (v1.13.10) (37). Alignment to a reference genome was conducted with Bowtie 2 (v2.4.5) (38), followed by indexing using Samtools (v1.16.1) (39) and viewing by Integrative genome viewer (v2.13.0) (40).

Using feature count data, samples with reads in 8/8 pediatric B-ALL samples and samples with reads in 6/6 NCD samples were delineated. The list of transcripts was extracted and run through the Ghent Venn diagram packaged https://bioinformatics.psb.ugent.be/webtools/Venn/.

### 2.13 Bioinformatics

A miRNA-specific B-ALL EV signature (significantly differentially packaged miRNAs (DPMiRs) between NCD and B-ALL) was identified using sRNAtoolbox (sRNADE) (41) (accessed December 2022). Within the sRNAtoolbox analysis, five pipelines were used: DESeq, DESeq2, NOISeq, EdgeR and T-test. For the RNA agnostic analysis of differentially packaged RNAs (DPRNAs), read counts were obtained with featureCounts (v2.04) (42) in RStudio (v2022.02.3). DESEq2 in the R statistical environment (v3.4.1) was used to calculate the difference between the normalized read counts of RNA with the “relative log expression” (RLE) normalization method of Bioconductor package DESEq2 (v1.36.0) (43). Statistically significant differential packaging (DP) was determined using the false discovery rate correction (FDR) at a cut-off of 0.05 or lower, as indicated. The RNASeq data have been deposited in the NCBI Gene Expression Omnibus (GEO) under the accession number GSE239467.

RNA transcripts from the RNA agnostic pipeline were using Ensembl Biomart (44) into coding RNA (mRNA) and non-coding RNA (miRNA, tRNA, lncRNA, snoRNA, snRNA) and unclassified non-coding RNAs using NCBI (https://www.ncbi.nlm.nih.gov accessed on December 2, 2022), HUGO gene nomenclature committee (https://www.genenames.org accessed on December 2, 2022) and RNAcentral (https://rnacentral.org/ accessed on December 2, 2022).

Pairwise and multi-group differential expression analyses were performed using the R statistical environment (v3.4.1). For RNASeq data exploration and quality check, plot counts, principal component analysis (PCA), and volcano plots were generated using DESeq2 (v3.16) and ggplot2 (v3.4.0). To identify the pattern of genes linked to the identified DPRNAs and exclusively packaged genes, gene set enrichment analysis (GSEA) and pathway analyses were performed using clusterprofiler (v4.6.2) (45), tidygraph (v1.2.2), tweenr (v2.0.2), pathview (v1.46.1) (46), enrichplot (v1.18.0) and ggplot2 (v3.4.0). Unsupervised hierarchical clustering was performed on z scores of read counts that were regularized logarithmic transformation (rlog) by the DESEq2 platform, using the GENESIS software (v1.8.1) (47). Gene ontology (GO) and Kyoto encyclopedia of genes and genomes (KEGG) analyses were carried out using Cytoscape (48).

To identify the potential competitive endogenous RNA networks, the miRNA:mRNA, miRNA:lncRNA and miRNA:lncRNA interactions of annotated RNA subtypes were identified using miRcode (http://www.mircode.org accessed on December 9, 2022), miRwalk (http://mirwalk.umm.uni-heidelberg.de accessed on December 9, 2022), targetscan (https://www.targetscan.org/vert_80/ accessed on December 9, 2022), lncRRIsearch (http://rtools.cbrc.jp/LncRRIsearch/ accessed on December 12-18, 2022). ceRNA networks were constructed using Cytoscape (48), and the connecting RNA extracted into a table.

### 2.14 In-silico target gene prediction

Target gene predictions for DPmiRs were carried out using target gene prediction software miRDB version 6.0 (49, 50) and Target scan version 8.0 (51). miRDB predicts targets based on the miRDB MiRTarget algorithm, which predicts targets based on 3’UTR and seed sequence match. Predicted targets were ranked, including using target prediction scores. The predicted targets between both software were compared, and only those identified using both were taken as targets.

### 2.15 Additional statistical analysis

The statistical significance of differences in EV size and concentration between NCD and B-ALL was determined using an unpaired t-test using GraphPad Prism (v9.5.1).

## 3 Results

### 3.1 Pediatric B-ALL plasma contains more extracellular vesicles than NCD plasma

**Figure 2:**
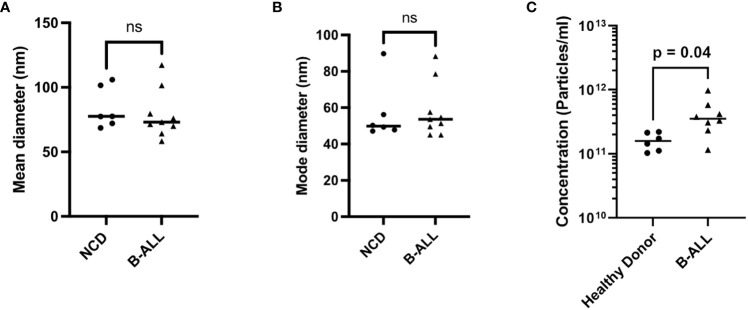
Characterization of EVs in non-cancer donors (NCD) and pediatric B-ALL blood plasma using nanoparticle tracking analysis showed a higher EV concentration in B-ALL. **(A)** Mean diameter, **(B)** mode diameter, and **(C)** concentration of plasma from NCD and pediatric B-ALL plasma. Statistical significance determined by unpaired T-test, ns: not significant.

We first used nanoparticle tracking analysis (NTA) to determine if the size or concentration of EVs differed in the PB plasma of newly diagnosed pediatric B-ALL (n=8, 3 *ETV6::RUNX1*, 3 *BCR::ABL1*-like) and NCD (n=6) at diagnosis. For NCD, the mean diameter is 55.2 nm +/- 15.8 nm (min 68.5 nm, max 105.9 nm) and for B-ALL the mean diameter is 58.9 nm +/-18.7 nm (min 58.3 nm, max 117.3 nm) (**Figures 2A**; **S1**). For NCD, the mode diameter is 82.7 nm +/- 16.5 nm (min 47.1 nm, max 89.7 nm) and for B-ALL is 74.3 nm +/- 15.1 nm (min 45 nm, max 88.3 nm) (**Figures 2B**; **S1**). A comparison of the mean and mode diameters (pediatric B-ALL versus NCD) showed that there is no statistically significant difference between NCD and pediatric B-ALL EV sizes (**Figures 2A, B**). A comparison of the EV concentration (number of particles/ml) of the NCD samples showed that NCD EV concentrations are almost uniform (**Figure 2C**). In contrast, B-ALL EV concentrations showed high sample-to-sample variability, suggesting that the pediatric B-ALL samples are highly heterogenous (**Figure 2C**). Overall, pediatric B-ALL PB plasma has a significantly higher EV concentration than NCD (**Figure 2C**).

### 3.2 Vn96 isolates a broad spectrum of EVs

In the absence of a gold standard EV isolation technique that is clinically amenable for EV analysis, we first evaluated commercially available EV isolation techniques to identify the best method for our study. For our purposes, the criteria for clinical amenability include having an easy protocol with applicability in the clinic, compatibility with small volumes (typically <2 ml), good EV yield, low co-isolation of contaminating proteins, and not requiring any specialized equipment. Using available literature and previous experience in our lab, we identified size exclusion chromatography (SEC) and polyethylene glycol (PEG) polymer-based ExoQuick as increasingly popular isolation techniques suitable for our purposes (52). We also identified the synthetic peptide Vn96 as appropriate for our purposes due to the ease of use and previous experience (31).

First, we isolated EVs from the conditioned culture media (CCM) of pediatric B-ALL cell lines RCH-ACV and UoC-B1, followed by Western blot analysis to characterize the EVs. We probed for EV-specific markers - tetraspanins CD63 and CD81 and heat shock proteins HSC70 and HSP90; B cell marker BLNK; and the contamination marker Calnexin (endoplasmic reticulum marker). The results showed that all three isolation methods isolate EVs from CCM but with different cargo (**Figures S2A–C**). Specifically, ExoQuick (**Figure S2A**) and SEC (**Figure S2B**) isolate EV subpopulations from pediatric B-ALL CCM that are enriched for CD63 or CD81, respectively. In contrast, Vn96 isolates a more comprehensive EV population, as shown by the inclusion of multiple EV markers, including CD63, CD81, HSP70 and HSP90 (**Figure S2C**). EVs were also isolated from healthy donor plasma and probed for the above-described EV markers, as well as CD41 (platelet activation marker) and markers of contamination for plasma: Albumin, Apolipoproteins A (High-density lipoprotein), Apolipoprotein B (low-density lipoprotein) and CD235a (Erythrocytes). EVs isolated from plasma using Vn96 showed a similar protein marker profile as the Vn96-isolated EVs from CCM, with a low signal for plasma contamination markers (**Figure S2D**). Finally, the Vn96 isolated EVs have a round morphology, showing a characteristic double membrane (53) (**Figure S3**). Therefore, we selected Vn96 for subsequent EV isolations.

### 3.3 NCD and pediatric B-ALL EVs package all types of small RNA

**Figure 3:**
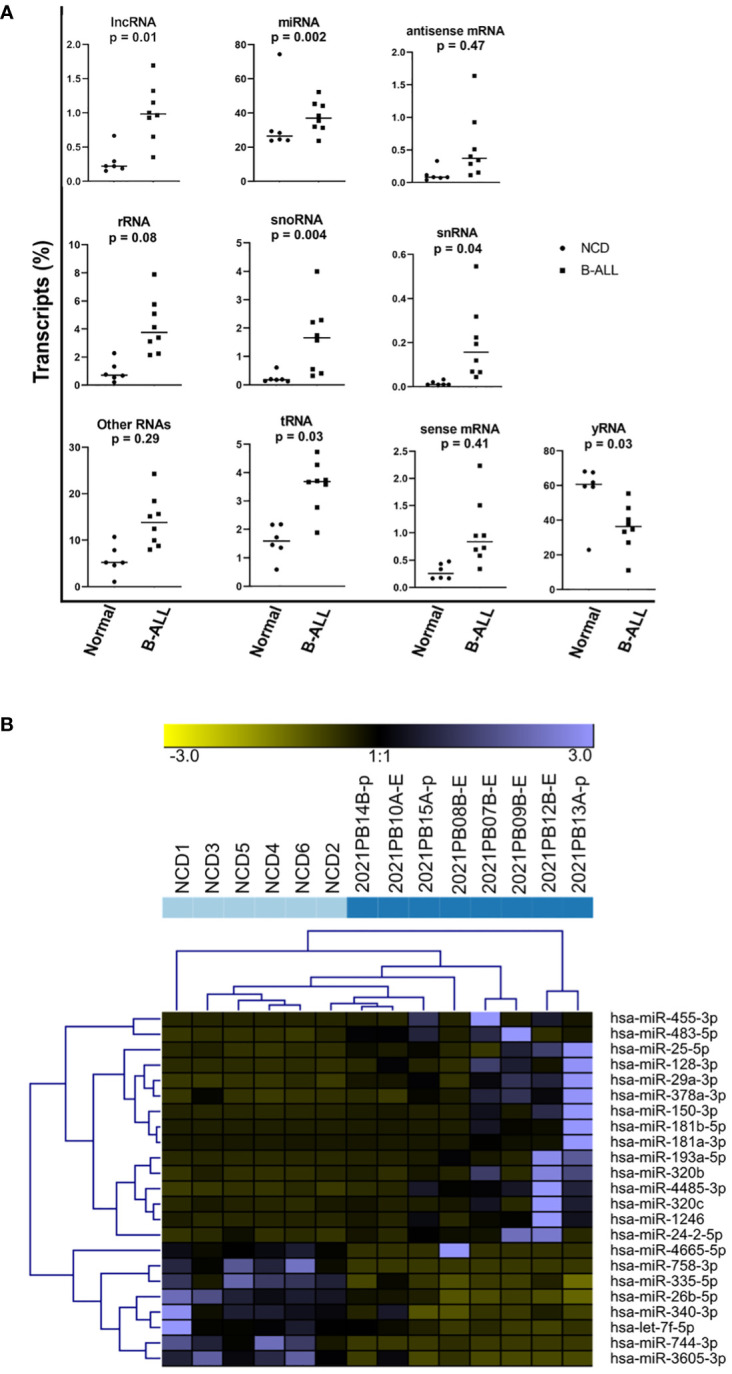
A microRNA (miRNA)-specific EV signature does not distinguish pediatric B-ALL from non-cancer donors (NCD). Raw small RNA-Seq data was analyzed using the sRNADE platform (SRNA toolbox). **(A)** Eleven RNA transcript species groups packaged into NCD and pediatric B-ALL plasma EVs were identified. Significance determined by t-test. **(B)** Unsupervised hierarchical cluster analysis using the sRNADE platform and identified differentially packaged miRNA signatures (FDR <0.05).

We next performed small RNA sequencing analysis (small RNA-seq) on RNA extracted from EVs that were isolated from B-ALL plasma compared to NCD plasma. sRNADE-based annotation of the RNA transcripts present in NCD and pediatric B-ALL EVs revealed that many different types of RNA transcripts are packaged into EVs with 11.3% +/- 7.8% unassigned (**Figure 3A**; unassigned not shown). Packaged RNA transcripts include miRNAs, lncRNAs, tRNAs, mRNAs, and snRNAs. The most abundant types of RNA transcripts are miRNAs and fragments of yRNAs. miRNA made up a minority of the total RNA in most cases and they become the majority only after excluding the unassigned transcripts. We found that variable miRNA transcript distribution between NCD and pediatric B-ALL exists (**Figure 3A**). The difference in abundance is statistically significant for lncRNA (p=0.01), snoRNA (p=0.004), snRNA (p=0.04), tRNA (p=0.03) and yRNA (p=0.03), with only yRNA decreased in B-ALL and all other RNA species increased. Closer inspection of the RNA transcripts suggests that many are fragments, as is consistent with the isolation of small RNA, which was verified by the analysis on the Agilent fragment analyzer.

### 3.4 A miRNA-specific signature does not clearly distinguish NCD from pediatric B-ALL

The differentially packaged miRNAs (DPmiRs) between NCD and B-ALL EVs were analyzed using the miRNA-specific sRNADE pipeline. DPmiRs (FDR<0.05) that were significant with all five platforms were analyzed by unsupervised hierarchical cluster analysis (**Figure 3B**). miRNAs downregulated in B-ALL EVs include miR-758-3p, miR-335-5p, miR-26b-5p, miR-340-3p, and let-7f-5p, while some miRNAs were upregulated in one or two B-ALL samples. Both mature and precursor miRNAs were detected in all samples (**Table 2**). However, analysis of these transcripts did not clearly distinguish NCD from B-ALL samples (**Figure 3B**).

**Table 2:** Total detected miRNA transcripts.

Sample	Detected miRNAs	Percent detected miRNAs	Detected precursor sequences	Percent detected precursor sequences	Total
NCD1	500	57	379	43	879
NCD2	670	56	517	44	1187
NCD3	832	58	610	42	1442
NCD4	578	57	433	43	1011
NCD5	714	57	531	43	1245
NCD6	470	57	354	43	824
2021PB07B_E	347	56	278	44	625
2021PB08B_E	498	56	386	44	884
2021PB09B_E	296	55	242	45	538
2021PB10A_E	509	57	385	43	894
2021PB12B_E	300	55	246	45	546
2021PB13A_p	427	56	332	44	759
2021PB14B_p	462	56	364	44	826
2021PB15A_p	329	56	258	44	587

### 3.5 Sample heterogeneity of plasma EVs

**Figure 4:**
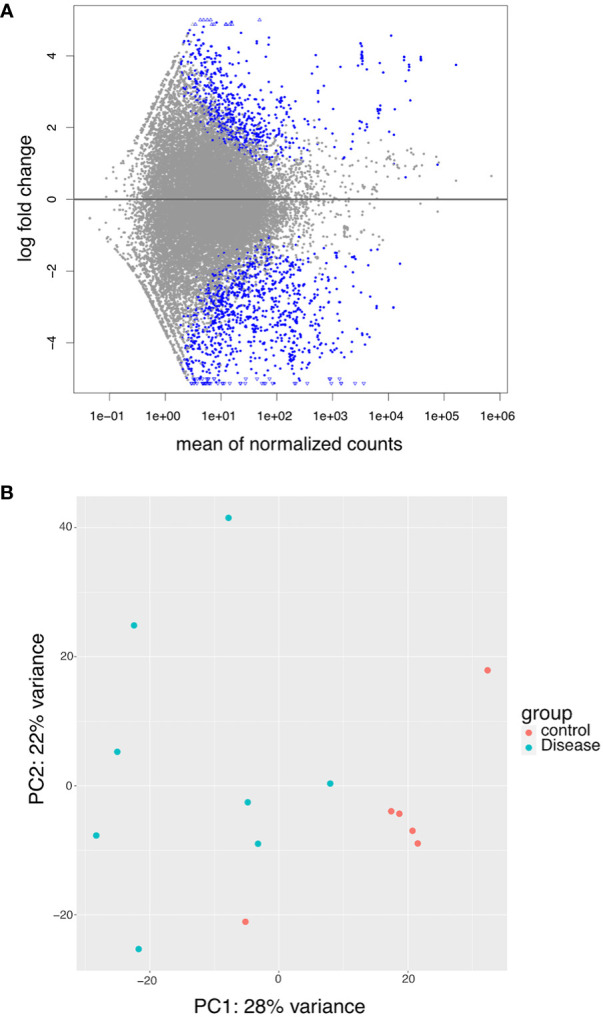
RNASeq data exploration. **(A)** MA plot showing differential packaging of sequences in B-ALL and NCD. Each dot represents one gene, blue are statistically different (FDR<0.05). Gens above the line are increased in B-ALL and genes below the line are decreased in B-ALL. Triangles indicate outliers. **(B)** PCA plots showing clusters of samples based on their similarities. Principal component 1 (PC1) is shown on the x-axis and PC2 on the y-axis. The legend is shown on the right – pink circle – NCD and green circle – B-ALL samples.

As there was a heterogenous mixture of small RNA detected, we next analyzed the small RNA-seq data in an agnostic manner (i.e., analysis of all transcript types). MA plots, a scatterplot of average expression signal (A -x axis) and log2 fold change (M – y axis), showed a higher distribution of points below 0 on the y-axis, suggesting a higher number of genes with decreased packaging than increased packaging (**Figure 4A**). The principal component analysis showed that most NCD (control) EVs clustered closely together (**Figure 4B**). In contrast, B-ALL (disease) EVs tended to have greater variance on the PCA2 axis, demonstrating clear heterogeneity between patients.

### 3.6 EV RNA transcripts discriminate B-ALL from NCD

**Figure 5:**
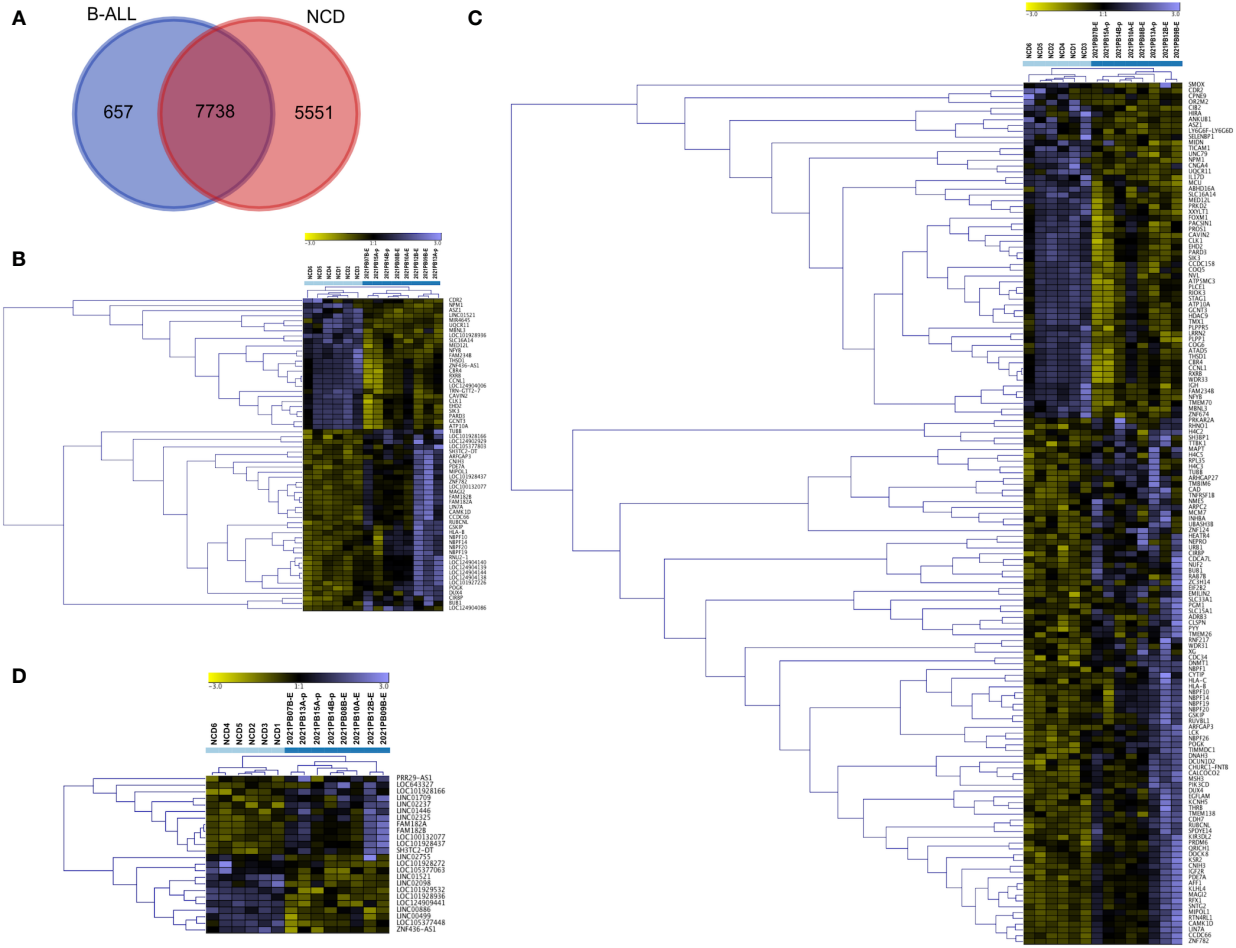
Analysis of small RNA using DESeq2 distinguishes pediatric B-ALL from NCD. All differentially packaged RNA (DPRNAs) were manually categorized and underwent unsupervised hierarchical cluster analysis based on Z-scores. **(A)** Venn diagram showing comparison of samples with reads in 8/8 pediatric B-ALL samples and 6/6 NCD, **(B)** unsupervised hierarchical cluster for RNA agnostic signature (FDR <0.0001), **(C)** unsupervised hierarchical cluster for mRNA with FDR<0.05, **(D)** unsupervised hierarchical cluster for long non-coding RNA (lncRNA) with FDR<0.05. Light blue squares are for NCD, and dark blue squares is for pediatric B-ALL.

We then compared all transcripts that were detected in B-ALL. We found that 7738 transcripts were present in both states (**Figure 5A**). We explored these transcripts further to determine if they are differentially packaged in B-ALL EVs compared to NCD EVs. Using DESEq2, we identified differentially packaged small RNA (DPRNA) that includes mRNAs, lncRNAs, tRNA, and snRNAs (**Figure 5B**; **Supplementary File 1**). A gene signature was clear when the top DPRNAs (FDR <0.0001) were viewed using unsupervised hierarchical clustering analysis (**Figure 5B**). The clustered transcripts include lncRNAs and mRNAs with only one miRNA transcript (miR-4645) making the cut-off. We did not find any discrimination based on sub-type as *ETV6::RUNX1* and *BCR::ABL1*-like samples were found in both B-ALL clusters.

We then manually annotated and divided the DPRNA signature (FDR<0.05) into different types of RNA transcripts, followed by unsupervised hierarchical cluster analysis. The mRNA fragments-based signatures included RNA from intronic regions such as NEPRO, EHD2, and RFX1 and from exons such as WDR31, NBPF10, NBPF14, NBPF19, NBPF20 and HLA-B. For the cluster of differentially packaged mRNAs (DPmRNAs), NCD and B-ALL formed two clear independent clusters (**Figure 5C**; **Supplementary File 1**). All NCD samples except NCD6 were very similar. Conversely, the B-ALL clade showed four subclades. Genes with increased packaging in NCD EVs include MED12L, MIDN, ASZ1, and IGH mRNA while genes that are increased in B-ALL include NBPF-10, -14, -19 and 20, and HLA-B and -C (**Figure 5C**). lncRNA fragments in the signature included long intergenic regions (lincs) such as LINC01521 and LINC02237, antisense regions such as ZNF436-AS1 and divergent transcripts such as SH3TC2-DT. The cluster of lncRNAs showed two main clades by sample, again with NCD clustering away from B-ALL (**Figure 5D**; **Supplementary File 1**). DPSpliceosomal RNA (snRNA), DPsnoRNA, DPmiR, DPtRNA, and unclassed RNAs with FDR<0.05 did not discriminate NCD from pediatric B-ALL (**Figure S4**). Overall, the DPRNA subtypes have different potentials for discriminating B-ALL from NCD; however, the total small RNA signature is very clearly discriminatory between NCD and pediatric B-ALL (**Figure 5B**).

**Figure 6:**
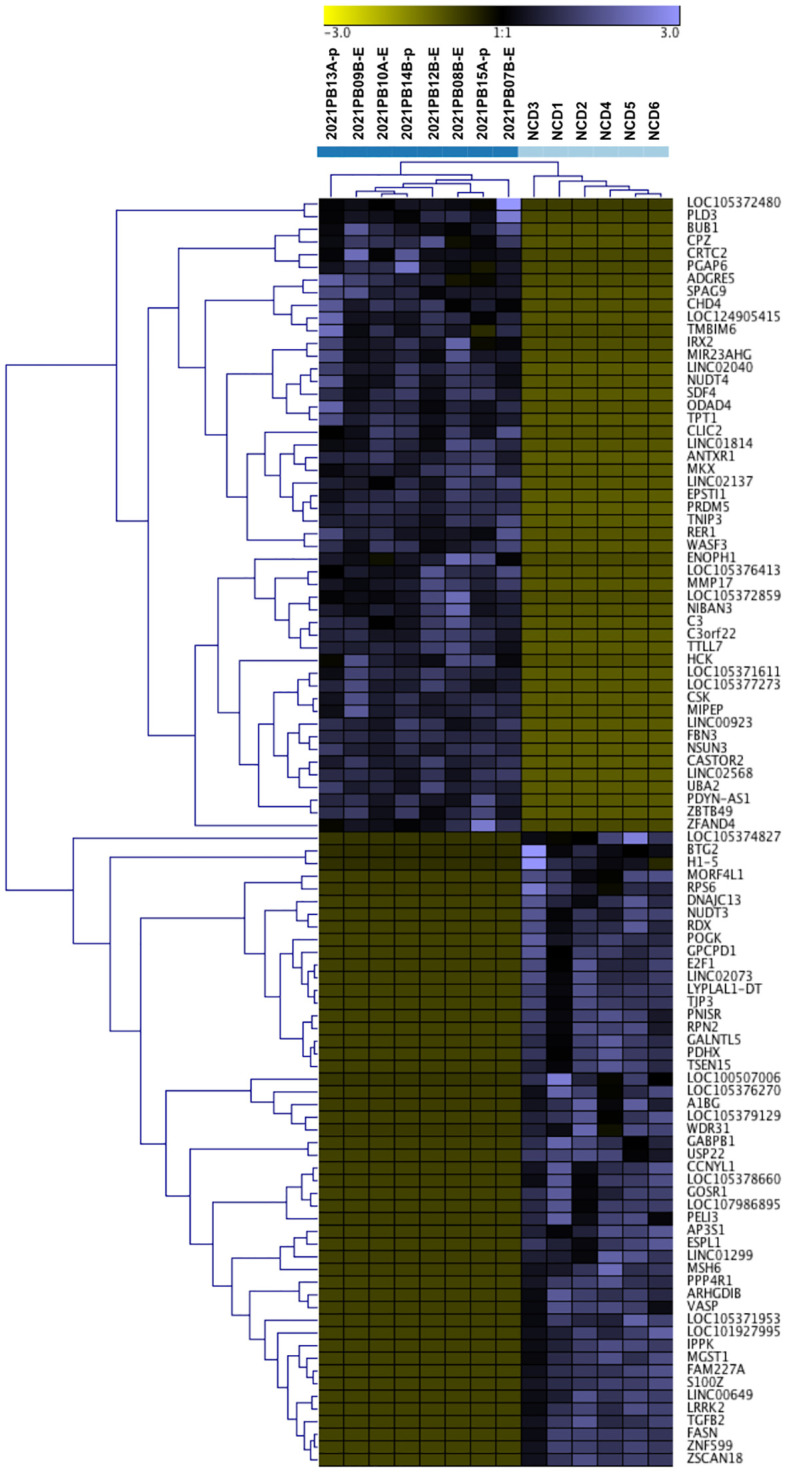
Analysis of small RNA exclusive to B-ALL and NCD is highly discriminatory. Unsupervised hierarchical cluster of the z scores of relative log transformation of top 100 RNAs exclusive to pediatric B-ALL and NCD samples respectively. Light blue squares are for NCD, and dark blue squares is for pediatric B-ALL.

We next identified genes exclusive to 8/8 B-ALL samples and found that 657 RNA transcripts have reads in all B-ALL samples with no detection in NCD (**Figure 5A**; **Supplementary File 2**). Similarly, 5551 RNA transcripts are exclusive to all NCD samples (6/6) (**Figure 5A**; **Supplementary File 2**). Each group’s top 100 RNA transcripts, sorted by average expression, were grouped and Z-scores calculated for hierarchical cluster analysis. As expected, the B-ALL exclusive and NCD exclusive transcripts show a clear discriminatory pattern between B-ALL and NCD (**Figure 6**). Thus, two sets of discriminatory EV RNA signatures were identified. One is a signature indicative of the normal control donor RNA profile, whereas the second signature consists of pediatric B-ALL exclusive RNAs (present in all 8 of our pediatric B-ALL samples) that represent the disease state.

### 3.7 Both fragments and whole transcripts are packaged into EVs

**Figure 7:**
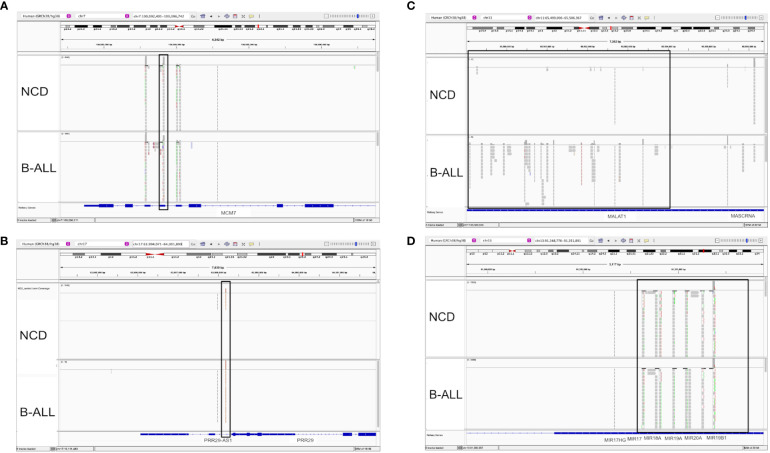
Visualization of RNA-seq data from selected DPRNAs using an integrative genome viewer (IGV) showed different RNA packaging patterns. IGV images comparing a non-cancer donor (NCD, top section of each panel) and pediatric B-ALL patient sample (bottom section of each panel) were visualized. The top toolbar shows the specific location address of the transcript and relative position on the chromosome. The top portion of each track shows the relative abundance in log scale while the lower portion of each track show the specific fragment sequenced. **(A)** A representative mRNA transcript, *MCM7*, with eight exons and eight intronic regions is shown. The black box highlights differential packaging of exon 2, with lower levels in B-ALL. **(B)** A representative long coding RNA (lncRNA) transcript, *PRR29-AS1*, contains five exons and four introns. The black box highlights that a transcript from intronic region two is differentially packaged between B-ALL and NCD, with higher levels in B-ALL. **(C)** A representative lncRNA transcript, *MALAT1*, is shown with a single exon. The black box highlights that multiple fragments of *MALAT1* lncRNA being more highly packaged into B-ALL EVs than NCD. **(D)** Several representative microRNAs (miRNAs) (from left to right: *miR-17HG*, *miR-17*, *miR-18A*, *miR-19A*, *miR-20A*, and miR-19B1) shown encoded on a single continuous coding region on chromosome 13 are packaged into NCD and B-ALL at relatively the same levels.

Different small RNA species and regions were found to be packaged into the EVs. Only exonic regions are packaged into EVs for some genes, for example, DPmRNA MCM7 (**Figure 7A**). Unexpectedly, the introns of other genes were found to be packaged into EVs, such as DPlncRNA PRR39-AS1 (**Figure 7B**). MALAT1, a lncRNA of 8779 nucleotides, had multiple fragments of the single exon gene packaged into EVs (**Figure 7C**), while multiple miRNA genes on a single exon are packaged into EVs (**Figure 7D**). These data suggest that full transcripts of some RNA sequences such as miRNA are packaged into EVs. However, the potential that whole lncRNA or mRNA transcripts are packaged needs to be explored further before more concrete conclusions can be reached.

### 3.8 Gene set enrichment analysis (GSEA) shows that B-ALL EVs package gene sets that negatively regulate the cell cycle

**Figure 8:**
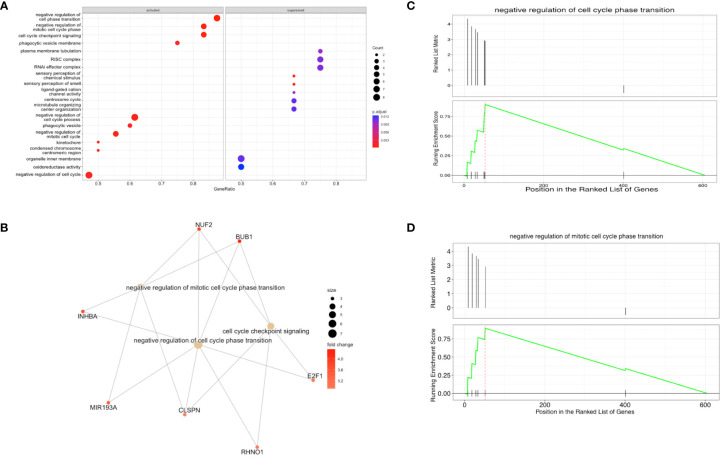
Gene set enrichment analysis (GSEA) shows that transcripts that negatively regulate the cell cycle are enriched in B-ALL EVs **(A)** Enriched and suppressed pathways, with the gene ratios shown on the x-axis and the associated biological process on the y-axis. The lefthand side of the plot shows gene sets that are overrepresented in B-ALL EVs (activated pathways) and righthand side of the plot shows genes sets that are excluded from B-ALL EVs (suppressed pathways). **(B)** enriched gene sets and known associated RNA species (orange circles – mRNAs or miRNA, yellow circles – gene sets) **(C, D)** Enrichment plots where the upper half is the ranked list metric, showing the ranking of the gene set in the list, and the lower half is the running enrichment score, including the position in the ranked list of genes. A total of 605 genes were annotated and analyzed.

Using GSEA, we found that gene sets within the genes both exclusive to B-ALL and DPRNAs are preferentially packaged into EVs (activated). These gene sets include negative regulation of cell cycle processes, checkpoint signaling, response to stress, and cellular and biological processes (**Figure 8A**). The genes associated with the identified gene sets are shown in relation to the associated gene sets (**Figure 8B**). This illustrates that many of the genes are associated with processes related with the overarching theme of negative regulation of cell cycle (**Figure 8B**). Genes such as CLSPN and BUB1 are linked to negative regulation of cell phase transition, while miR-193A is linked to negative regulation of mitotic cell cycle phase transition. Representative plots of specific gene sets exemplify the high enrichment scores for “negative regulation of cell cycle” and “negative regulation of mitotic cell cycle phase transition” (**Figures 8C, D**).

### 3.9 GO and KEGG pathways of exclusive signature

**Figure 9:**
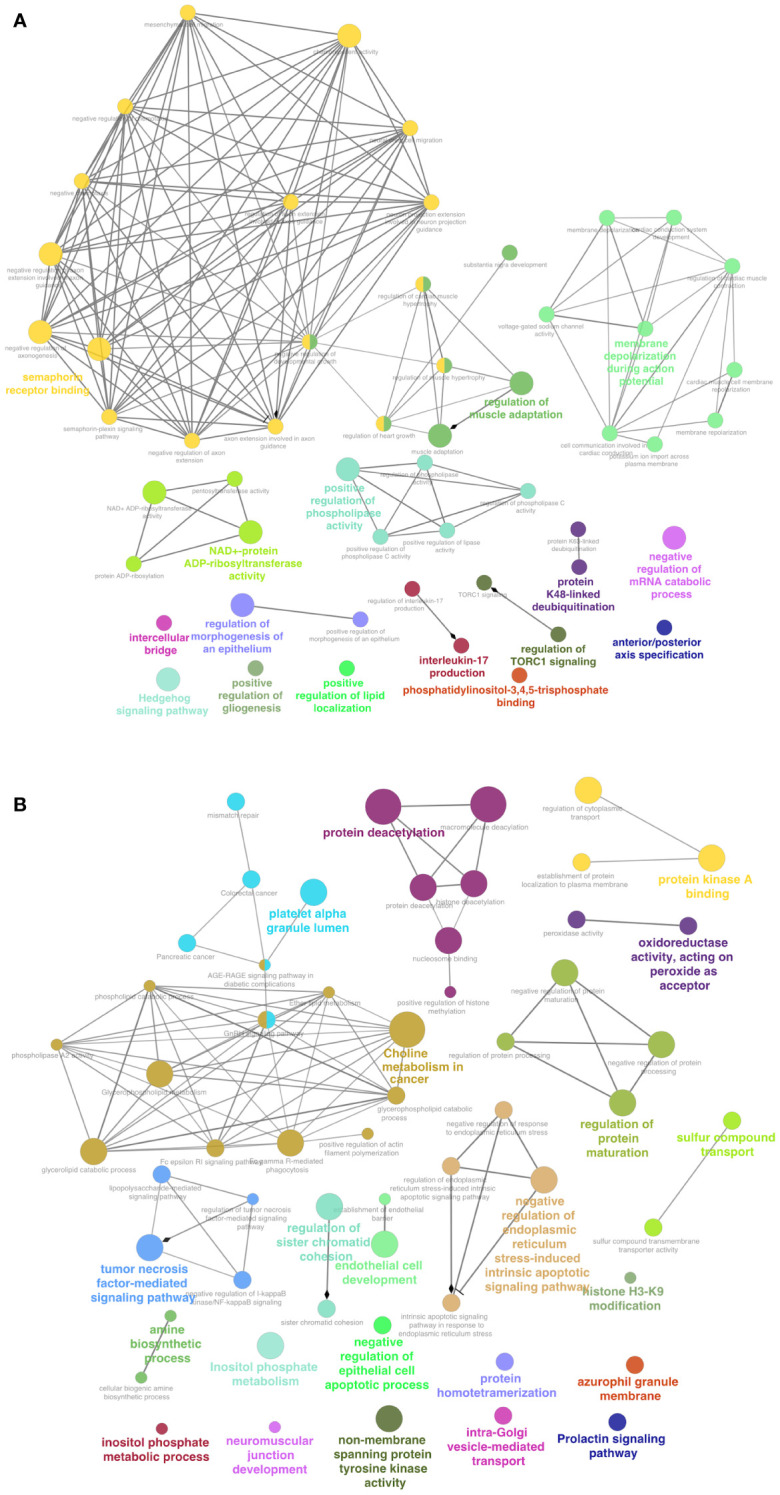
Functions enriched in NCD represent multiple cell types, while functions in B-ALL are related to cancer metabolism. Gene ontology (GO) and Kyoto Encyclopedia of exclusive RNA genes and genomes (KEGG). **(A)** Top 100 RNA transcripts exclusive to 6/6 NCD samples. **(B)** Top 100 RNA transcripts exclusive to 8/8 pediatric B-ALL samples. The networks were visualized using Cytoscape. P<0.05 for all enrichments.

The GO and KEGG analysis of the top 200 NCD-exclusive EV RNAs showed key processes including semaphorin binding, intercellular bridge, interleukin 17-production and regulation of TORC1 signaling (**Figure 9A**; **Supplementary File 3**). Other pathways include protein maturation regulation, histone H3-K9 and neuromuscular junction development. Conversely, the top 200 pediatric B-ALL-exclusive EV RNAs showed pathways such as tumor-necrosis factor mediated signaling, choline metabolism in cancer, inositol phosphate metabolic process, platelet alpha granule lumen processes and protein deacetylation (**Figure 9B**; **Supplementary File 4**). These data suggest that sequences from multiple cell types are packaged in NCD EVs while more cancer specific genes are packaged in B-ALL EVs.

### 3.10 Competitive endogenous RNA (ceRNA) analysis shows miRNA:mRNA pairs

To identify the ceRNA network associated with the total DPRNA signature, we identified miRNA: lncRNA and miRNA:lncRNA interactions using lncRRIsearch and targetscan. We identified miRNA:mRNA interactions using miRcode and miRwalk. We found lncRNA:mRNA, pairs instead of miRNA: lncRNA:mRNA triplicates (**Table 3**). This is not totally unexpected, as the ceRNA field is currently in its infancy, especially in pediatric B-ALL, and the interaction networks are based on what has been previously published.

**Table 3:** List of potential long non-coding RNA (lncRNA): messenger RNA (mRNA) pairs of competitive endogenous RNA interactions for differentially packaged RNA transcripts.

	lncRNA	mRNA target
1	LINC02325	ASZ1
2	ZNF346-AS1	BUB1
3	SH3TC2-DT	ATP5MC3
4	PRR29-AS1	ATP10A
5	LINC02755	ATAD5
6	LINC00499	ABHD16A
7	LINC02237	ARPC2
8	LINC02098	ARHGAP27
9	LINC01709	ARFGAP3
10	LINC01521	ANKUB1
11	LINC01446	AFF1
12	LINC00886	ADRB3

**Figure 10:**
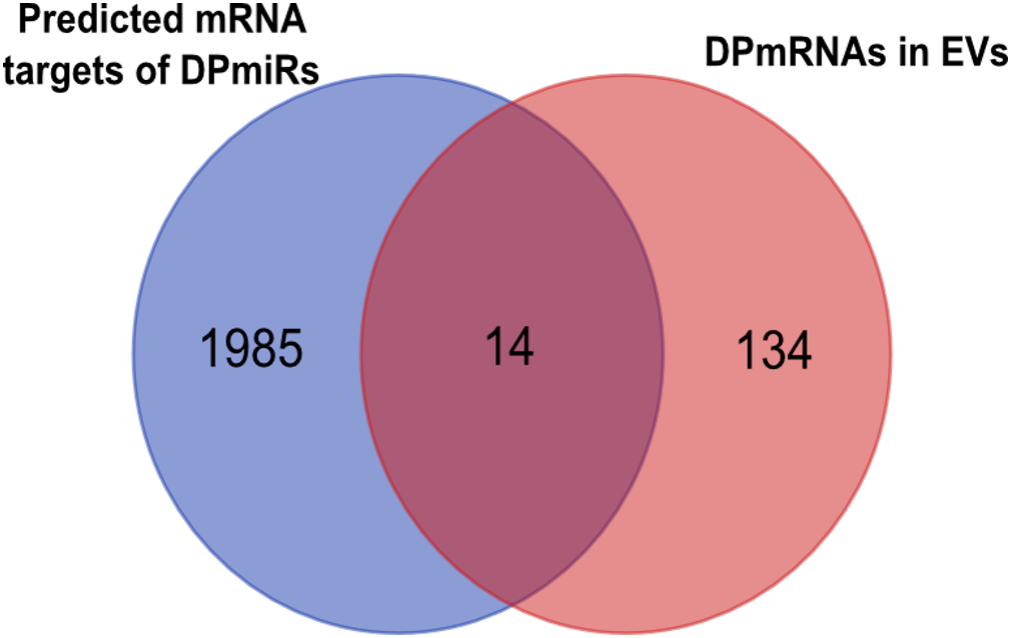
microRNA (miRNA) and their targets are packaged into EVs, as shown in the Venn diagram showing mRNA targets of differentially packaged miRNA and mRNA cargo of EVs.

The targets of the 4 DPmiRNAs from the RNA agnostic pipeline were predicted using two different platforms and overlapping target mRNA between both platforms used for visualization of miRNA:mRNA interaction networks (**Supplementary File 5**). Predicted mRNA targets were compared to DPmRNAs (**Figure 10**), showing that there are 148 DPmRNAs, and 1999 mRNA targets, of which 14 mRNA are overlapping between the predicted mRNAs and packaged mRNAs. These are ATP10A, RNF217, MBNL3, MED12L, CDH7, MIPOL1, NFYB, AFF1, SLC15A1, MAGI2, KCNH5, XG, CDC34, KSR2. Overall, our data suggests that the different RNA transcripts differentially packaged into pediatric B-ALL EVs interact with each other in varying ways that are not yet fully understood.

## 4 Discussion

In this pilot study, we found that EVs from two types of B-ALL PB plasma at diagnosis have a similar size profile to NCD PB EVs. This size range suggests that the EVs in both groups of samples could be either larger exosomes and smaller microvesicles. With reference to EV numbers, the overall concentration of EVs was higher in B-ALL than NCD BP. This finding aligns with what has previously been discovered in other cancers (54, 55), including in AML (56) and CLL (57). We then used the clinically amenable Vn96 synthetic peptide to isolate EVs from BP samples and subsequently, identified a signature of EV small RNAs that is exclusive to NCD and B-ALL, as well as RNA sequences that are differentially packaged. The small RNAs found in these sub-types of B-ALL EVs tend to be overrepresented with genes that negatively regulate the cell cycle, suggesting that EVs may be used to discard RNA sequences that are inhibitory to B-ALL growth and progression. In contrast, NCD EVs have a mix of different transcripts that appear to originate from multiple organs, including the central nervous system, muscle, platelets, and epithelial cells.

Consistent with our NTA findings, the Boyiadzis group quantified extracellular vesicles (by protein content) of sera from normal controls and AML patients, and found a higher EV concentration in AML EVs than normal controls (56). Furthermore, targeted protein profiling of leukemia-associated antigens CD34 and CD117 in AML EVs versus control EVs showed differential representation of these markers, which were higher in AML than controls (56). Using a different cohort of donors and samples, the Boyiadzis group used TGF-ß1 levels to track EV concentration, and found that AML EV burden is reduced post-chemotherapy induction, increased during consolidation, and normalized during complete remission, suggesting that AML EVs reflect leukemia burden (58). Similarly, in B-cell CLL, the EV concentration was higher in patient blood plasma than in normal controls (59). Furthermore, CLL EV concentration is reduced post-therapy (57). Thus, plasma EV concentration may be a surrogate marker for monitoring B-ALL disease progression and response to therapy; however, demonstrating this will require validation in a larger cohort of samples that includes post-treatment samples.

A key aspect of EV biology that determines their functionality is their cargo. Specifically, EV RNA cargo has been shown to be very important for overall EV functionality and interactions with recipient cells and environments (60). Furthermore, EV cargo has been shown to facilitate cancer progression and disease etiology (61). Therefore, we decided to examine the small RNA cargo profile of pediatric B-ALL EVs, as a window into the disease. To identify the RNA profile and signature, we used the sRNADE platform (comprehensively called sRNAtoolbox) to annotate the RNA transcripts detected in the plasma EVs. Interestingly, both mature and precursor miRNA (pre-miRNA) transcripts were detected. While mature miRNAs have been widely reported to be packaged into EVs in different biofluids and diseases, including acute myeloid leukemia (62), pre-miRNAs have rarely been reported. However, tumor-derived microvesicles (TMVs) isolated from LOX melanoma cells carry pre-miRNAs such as pre-miR-21, pre-miR-27, pre-miR-100 and pre-miR-151 (63). Other transcript types that we also identified as packaged into EVs included non-coding RNAs (ncRNA) such as snRNA, snoRNAs, yRNAs and lncRNA. The distinction between intact versus fragmented was determined based on IGV visualization of the RNA transcripts, where we observed evidence for both. mirVana isolates total small RNA (<200 nt), but RNA transcript types such as lncRNAs and tRNAs are >200nt. Thus, the data suggest that both fragmented and intact mRNAs, tRNAs, and lncRNAs are packaged in EVs. While this is the first time that this has been reported in pediatric B-ALL, other research groups have previously identified similar RNA species as part of EV cargo in healthy and diseased cells (21, 64). In addition, some earlier publications reported that miRNAs are the most abundant RNA transcripts in EVs (19, 65); however, we and others (66, 67) have not reproduced this finding when unassigned transcripts are considered.

Examples of EV RNA sequences that are exclusive to all NCD samples include miR-12136, a regulator of translation (68), NOC2L, a regulator of histone acetyltransferase activity (69) and PERM1, a regulator of mitochondrial content and oxidative function (70). In addition, lncRNA LINC01134 directly binds the promoter of AKTISI, which in turn leading to activation of the NFKB signaling pathway (71). Another prevalent lncRNA, LINC01714, is known to suppress proliferation, migration and invasion in cholangiocarcinoma (CCA) cells (72). Combined, NCD-exclusive EV RNA transcripts appear to be associated with generic and varied cell types. This suggests that in normalcy, blood cells and cells with access to the bloodstream secrete EVs and contribute to the total plasma EV repertoire. Conversely, the EV RNAs exclusive to the B-ALL samples we investigated include C3, a complement protein, which is a serum effector of innate immunity. High C3 levels indicate inflammation, which may be associated with many hematological malignancies (73). Other top genes include ODAD4, which is associated with ALL (74), LINC02568, which is linked to squamous carcinoma (75) and MRPS31, which is a driver of carcinoma (76). Taken together, many of the B-ALL exclusive EV RNA transcripts are associated with disease, including hematological malignancies and immune activation.

Some of the mRNA that we found to be differentially packaged in this study are known to play active roles in leukemia and other cancers. mRNAs such as the LIN7A is differentially expressed in most samples and is known to be upregulated in AML (77), and PDE7A, which is upregulated in most B-ALL samples in our study, is known to be upregulated in CLL and is associated with elevated cAMP levels (78). Some known leukemia-associated transcripts such as DUX4 (79), IGH (80), NFYB (81) and MED12L (82) are notably differentially packaged, with DUX4 being more packaged and IGH, NFYB, and MED12L less packaged in the B-ALL EVs we studied. Some of the mRNAs with higher levels in B-ALL EVs are known to be pro-tumorigenic. For instance, MCM7, a marker of a high proliferation rate in cancer, is upregulated in B-ALL EVs (83). CDC34, which is upregulated in B-ALL EVs, is known to facilitate lung cancer progression (84), while NUF2, which is upregulated in the EVs of 6 of 8 B-ALL samples in this study, is known to facilitate the progression of lung adenocarcinoma (85). Finally, HLA-B and -C are suggested to be involved in tumorigenesis of different non-leukemia cancer types (86). Potentially, these pro-tumorigenic mRNAs may be increasingly packaged from the lymphoblasts in the bone marrow into B-ALL EVs because of their overrepresentation in B-ALL lymphoblasts.

We found that the DPmiRs identified using the RNA agnostic DESEq2 platform varied from the DPmiRs identified using the miRNA-specific platform sRNADE. This may be because of differences in the gene count data, as each platform uses a different algorithm for count data processing, which may skew the total counts in the miRNA specific versus RNA agnostic platforms. Unfortunately, the DPmiRs identified from the two different platforms (sRNADE and DESEq2) were not congruent or discriminatory enough for our purposes but matched previously known links to different cancers and in some cases, some types of leukemias, including B-ALL (87, 88).

LincRNAs, which characteristically do not overlap with protein-coding transcripts, give rise to intergenic long non-coding transcripts (89). So far, none of the lincRNAs we identified in this study are currently known to be involved with pediatric B-ALL or leukemia; however, some of the lincRNAs are linked to other cancers (90–92). Though a lot is not known about these lincRNAs in pediatric B-ALL, they may be potential targets for follow-up. Taken together, these DPlncRNAs may need to be studied further to ascertain their relevance or lack thereof, in pediatric B-ALL.

We further explored the significance of the exclusively packaged and differentially packaged RNA in B-ALL biology using GSEA. The gene sets activated/enriched include negative regulation of cell cycle checkpoint signaling. It is currently known that dysregulation of the cell cycle is a hallmark of B-ALL (93). The uncontrolled cell proliferation that results from cell cycle dysregulation could lead to leukemogenesis (94). Other gene sets activated/enriched in pediatric B-ALL EVs are phagocytic vesicle, kinetochore, and condensed chromosome centromeric region and organelle inner membrane. Overall, cell cycle and membrane-associated gene sets are activated (enriched). Conversely, gene sets such as membrane tubulation, RISC complex, oxidoreductase activity, and ligand-gated cation activity are suppressed (reduced). The GSEA is consistent with the hypothesis that packaging anti-tumour genes into EVs may drive an overactivation of pro-cancer pathways and processes to facilitate cancer tumorigenesis. Alternatively, the results of the GSEA may indicate increased expression of the DPRNAs in cells, leading to increased packaging of these RNAs into EVs. Gene sets that are excluded from B-ALL EVs are associated with oxidoreductase activity, organelle and plasma membrane organization, regulation of transcription, and signal transduction. Developing B cells, which start as hematopoietic stem cells (HSCs), need cellular redox homeostasis to maintain a dynamic balance needed for their normal proliferation (95), which begins in the bone marrow (BM) (96). The BM niche is hypoxic and generates reactive oxygen species (ROS) levels that are lower than in normal tissues, meaning that the BM niche is highly sensitive to changes in oxidative stress linked to oxidoreductase activity (97). Changes in oxidoreductase levels can lead to alteration of ROS levels, which can affect the HSCs, including developing B cells (98). Furthermore, moderate ROS acts as a second messenger that, in turn, regulates cell proliferation, while high ROS levels are linked to leukemic transformation (99). Thus, retention of these transcripts in the cell, as evidenced by reduced packaging in EVs, may promote the adaption of the cells to the BM microenvironment. Furthermore, the GSEA show themes that align with current knowledge about pediatric B-ALL onset, such as ligand-gated cation channel activity, cell cycle regulation and cell cycle phase transition (100, 101). These data suggest that the plasma EVs in B-ALL may preferentially package transcripts that impede proliferation, consistent with a role for EVs in the removal of unwanted transcripts; however, this remains to be proven via loss-of-function experiments to inhibit EV release and analyzing cell proliferation.

This study has some key limitations as we have only analyzed two different subtypes of B-ALL, *ETV6::RUNX1* and *BCR::ABL1*-like. Moreover, we have analyzed a limited number of samples, which further limits the findings. In addition, to have age-matched samples, we chose to use PB from children without cancer but have other disorders. This is because it is very difficult to ethically sample PB from healthy children. Therefore, additional study on healthy children would be an important step before these data could be moved into clinical use. Nevertheless, our data suggest that EVs in PB of patients with B-ALL differ from those that do not have cancer. Thus, further study is warranted on a larger validation cohort. In addition, analysis of EVs in a longitudinal manner to track EVs over the course of disease and treatment could allow clinicians to follow the disease in real time in a particular patient.

## 5 Conclusion

EVs play a role in shuttling signaling mediators between cells. In this study, analysis of the small RNA cargo of pediatric B-ALL EVs suggests that packaging of this cargo is not random and may be involved in aspects of disease onset and progression. We also showed the presence of a signature exclusive to all the EV RNA cargo of the NCD samples (normalcy), and a disease-associated signature that is exclusive to all B-ALL samples. Pediatric B-ALL EV concentration is also a potential biomarker for pediatric B-ALL, with increased EV levels that may precede the increase of cells in circulation. In this way, EV concentration could be a less invasive surrogate biomarker for monitoring disease burden, therapy response, and even early detection of MRD. Moving forward, a study to validate the findings from this study, using a larger patient cohort, with samples collected longitudinally and cross-sectionally is needed.

## Data availability statement

The datasets presented in this study can be found in online repositories. The names of the repository/repositories and accession number(s) can be found in the article.

## Ethics statement

The studies involving humans were approved by Health Research Ethics Board of the Health Research Ethics Authority of Newfoundland and Labrador. The studies were conducted in accordance with the local legislation and institutional requirements. The human samples used in this study were acquired from the BC Children's Hospital Biobank or as part of this study for which ethical approval was obtained. Written informed consent for participation was obtained from the participants or the participants’ legal guardians/next of kin in accordance with the national legislation and institutional requirements.

## Author contributions

ML: Conceptualization, Data curation, Formal Analysis, Investigation, Methodology, Writing – original draft, Writing – review & editing. J-AH: Conceptualization, Resources, Writing – review & editing. LP-C: Formal Analysis, Writing – review & editing. RC: Investigation, Writing – review & editing. BH: Investigation, Writing – review & editing. SC: Investigation, Writing – review & editing. SL: Investigation, Resources, Supervision, Writing – review & editing. PM: Conceptualization, Funding acquisition, Investigation, Supervision, Writing – review & editing. SLC: Conceptualization, Formal Analysis, Funding acquisition, Project administration, Supervision, Writing – original draft, Writing – review & editing.
