# Supplementary material for: Extracellular vesicle small RNA cargo discriminates non-cancer donors from pediatric B-lymphoblastic leukemia patients
Source: Front Oncol. 2023 Nov 13;13:1272883. doi: 10.3389/fonc.2023.1272883 (PMC10679349; doi:10.3389/fonc.2023.1272883)
Supplement: Supplementary file 1 [file DataSheet_1.pdf]

Supplementary Methods

1. *Polyethylene glycol-based isolation (ExoQuick)*

CCM was centrifuged at 3000 × g for 15 minutes to remove cells and cell debris. CCM supernatant was then incubated with ExoQuick TC precipitation reagent (System Biosciences, Palo Alto, CA) and incubated upright for at least 12 hours at 4°C. Post incubation, the ExoQuick TC/CCM mixture was centrifuged at 1,500 x g for 30 mins at RT, and the supernatant was removed. This was followed by centrifugation at 1,500 x g for 5 mins to remove any residual supernatant. Microsphere beads were prepared by washing in resuspension buffer before mixing with the beige/white EV pellet at room temperature (RT) on an inverting shaker for 15 minutes. Purified EVs were separated from the beads by centrifugation (8000 x g for 5 minutes at RT). The EVs were present in the supernatant, which was stored on ice for immediate use or at -80° C until ready for use.

2. *Size exclusion chromatography (SEC)*

CCM was centrifuged at 2,000 x g to pellet apoptotic vesicles and debris. IZON qEV2 size exclusion columns were removed from 4 °C, and 0.1µm filtered 1XPBS was allowed to run through the column. CCM was overlaid on the column, followed by elution with 0.1µm filtered 1X PBS. The flow-through was collected in 2 ml fractions (16); fractions 11 - 14 were combined for analysis.

3. *Western blot*

Primary Abs were diluted in 5% (w/v) skimmed milk in TBST as follows: 1:1000 HSP90a/ß (Santa Cruz, sc-13119), 1:1000 CD81 (Santa Cruz, sc166029), 1:1000 HSC70 (Santa Cruz, sc-7298), 1:500 CD63 (Santa Cruz, sc-5275), 1:500 Albumin (Cell Signaling Technology- cat no.4929), 1:10,000 Apolipoprotein B (Abcam, ab139401), 1:1000 Apolipoprotein A (Santa Cruz sc-376818), 1:1000 CD41 (Cell signalling technologies, CST 138075), and 1:1000 CD235 (Santa Cruz, sc-59182). HSP90, HSC70, ApoA, and CD63 were detected using HRP-conjugated goat anti-mouse IgG (Santa Cruz-13119), while ApoB, CD41, Calnexin and Albumin were detected using HRP-conjugated mouse anti-rabbit IgG (Santa Cruz, sc-2357). CD235 was detected using goat anti-rat IgG-HRP HRP conjugated (Santa Cruz, sc-2006). All secondary Abs were diluted 1:2000 in 5% (w/v) skimmed milk in TBST.

A

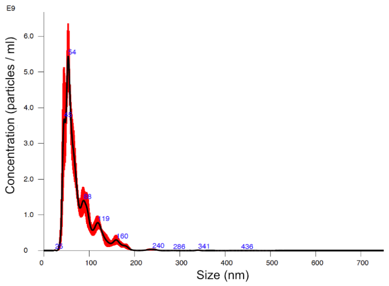

NCD 6
Mean diameter: 75.2 nm
Mode diameter: 53.6 nm
Concentration: 1.88 x 10^11(particles/ml)

B

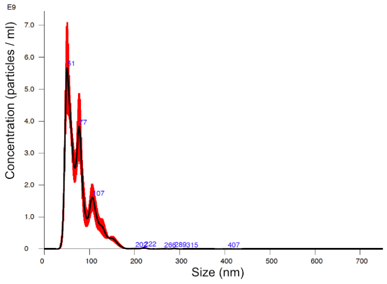

B-ALL 4311
Mean diameter: 77.4 nm
Mode diameter: 50.3 nm
Concentration: 2.21 x 10^11(particles/ml)

Figure S1. Sample NTA data for NCD and pediatric B-ALL samples, showing histogram – typical output of NS300 run.

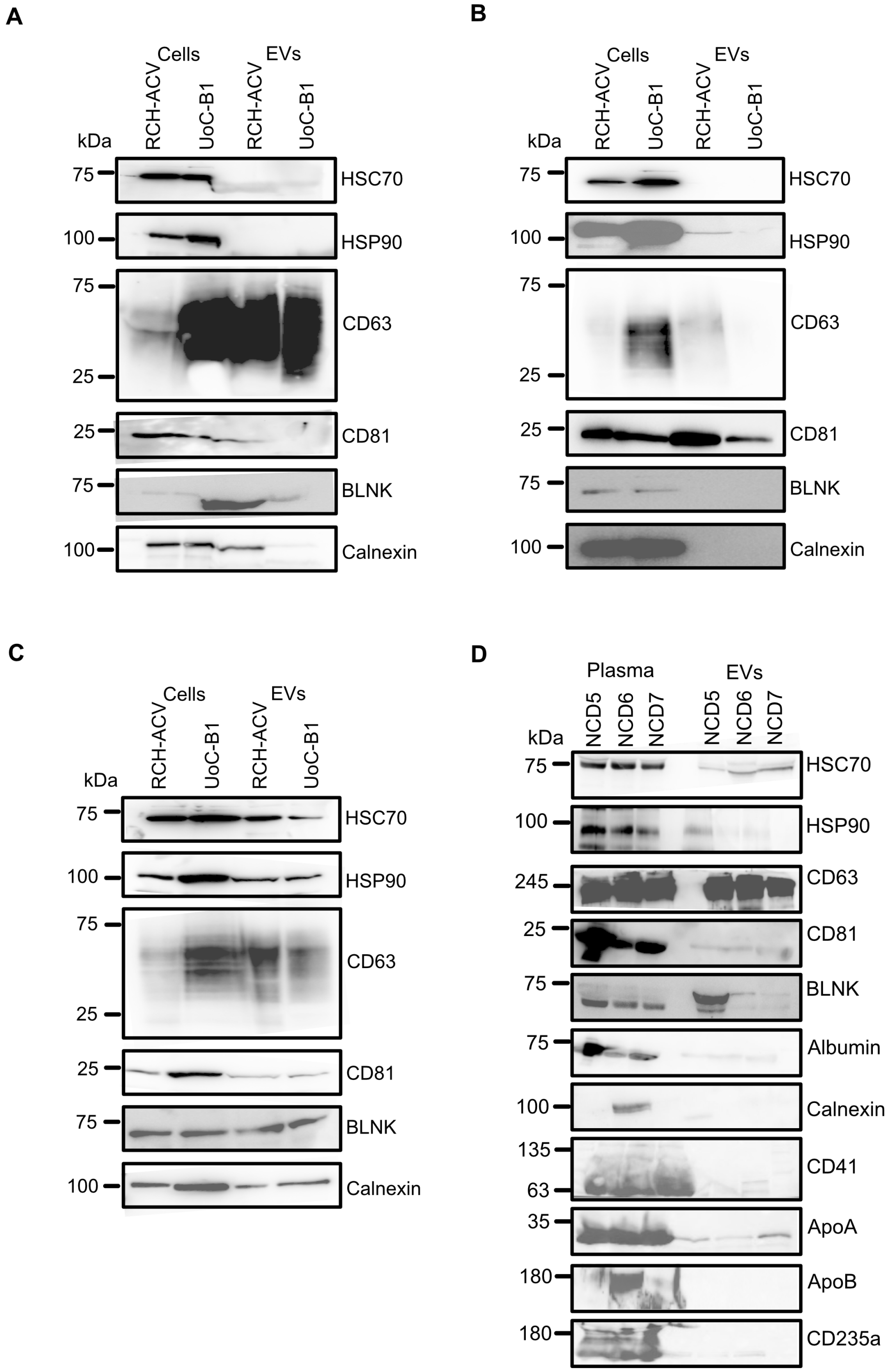

Figure S2. Western blot image comparing three EV isolation techniques (A) ExoQuick (B) SEC (C) Vn96 from cell line CM (D) Vn96 from NCD plasma. Molecular weight markers are shown on the left of each panel. EV markers: HSC70, HSP90, CD63, CD81; B cell marker: BLNK; non-EV markers: Albumin, Calnexin, CD41, ApoA, ApoB, CD235a.

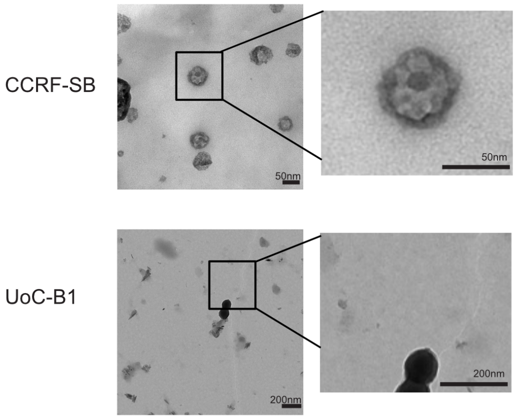

Figure S3. Transmission electron microscopy (TEM) images of pediatric B-ALL cell line EVs from CCRF-SB cell line (top) and UoC-B1 (bottom) isolated with Vn96.

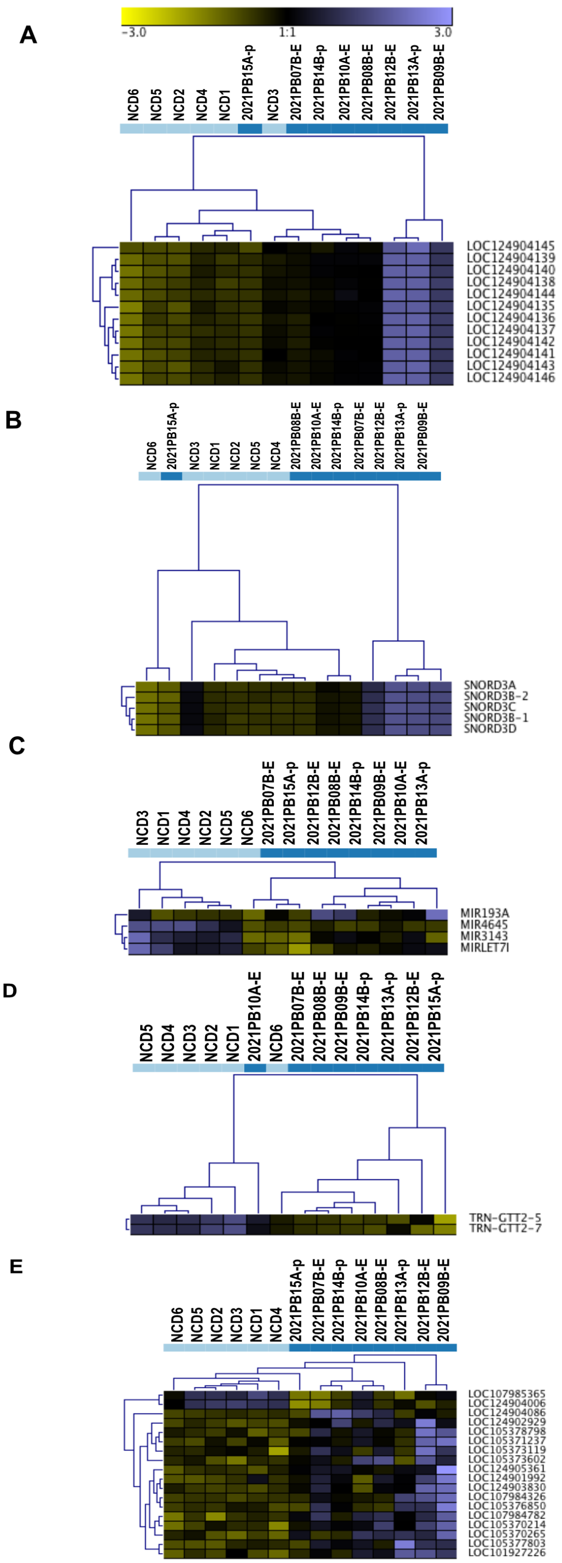

Figure S4. Unsupervised hierarchical cluster of small RNA EV clusters that are not clearly discriminatory with FDR<0.05. (A) spliceosomal RNAs (snRNA), (B) snoRNA, (C) miRNA (D) tRNA (E) unclassed. Light blue squares are for NCD, and dark blue squares are for pediatric B-ALL.
